# Disparities in incidence and survival for patients with Ewing sarcoma in Florida

**DOI:** 10.1002/cam4.7151

**Published:** 2024-04-23

**Authors:** Aditi Dhir, Rachna Rahul, Qinran Liu, Dan Pham, Rachel Kronenfeld, Tulay Koru‐Sengul, Paulo S. Pinheiro

**Affiliations:** ^1^ University of Miami Miller School of Medicine Miami Florida USA; ^2^ Division of Pediatric Hematology/Oncology, Department of Pediatrics University of Miami Miller School of Medicine Miami Florida USA; ^3^ Sylvester Comprehensive Cancer Center University of Miami Miller School of Medicine Miami Florida USA; ^4^ Division of Epidemiology & Population Health Sciences, Department of Public Health Sciences University of Miami Miller School of Medicine Miami Florida USA; ^5^ Division of Biostatistics, Department of Public Health Sciences University of Miami Miller School of Medicine Miami Florida USA

**Keywords:** Ewing sarcoma, Florida, health disparities, Hispanic

## Abstract

**Background:**

Ewing sarcoma (ES) is a malignant bone tumor most commonly affecting non‐Hispanic White (NHW) adolescent males, though recognition among Hispanic individuals is rising. Prior population‐based studies in the United States (US), utilizing Surveillance, Epidemiology, and End Results (SEER) have shown higher all‐cause mortality among White Hispanics, Blacks, and those of low socioeconomic status (SES). Florida is not part of SEER but is home to unique Hispanic populations including Cubans, Puerto Ricans, South Americans that contrasts with the Mexican Hispanic majority in other US states. This study aimed to assess racial/ethnic disparities on incidence and survival outcomes among this diverse Florida patient population.

**Methodology:**

Our study examined all patients diagnosed with osseous ES (2005–2018) in Florida (*n* = 411) based on the state's population‐based cancer registry dataset. Florida Age‐adjusted Incidence Rates (AAIRs) were computed by sex and race‐ethnicity and compared to the equivalent populations in SEER. Cause‐specific survival disparities among Florida patients were examined using Kaplan–Meier analysis. Univariable and multivariable analyses using Cox regression were performed for race/ethnicity, with adjustment for age, sex, year of diagnosis, site of disease, staging, SES, and insurance type.

**Results:**

There was a significantly higher incidence of osseous ES in Florida Hispanic males (AAIR 2.6/1,000,000); (95% CI: 2.0–3.2 per 1,000,000; *n* = 84) compared to the SEER Hispanic males (AAIR 1.2/1,000,000;1.1–1.4 per 1,000,000; *n* = 382). Older age, distant metastasis, lack of chemotherapy or surgical resection were statistically significant determinants of poor survival while SES, insurance status and race‐ethnicity were not. However, among nonmetastatic ES, Florida Hispanics had an increased risk of death compared to Florida NHW (adjusted Hazard Ratio 2.32; 95%CI: 1.20–4.46; *p* = 0.012).

**Conclusions:**

Florida Hispanic males have a higher‐than‐expected incidence of osseous ES compared to the US. Hispanics of both sexes show remarkably worse survival for nonmetastatic disease compared to NHW. This disparity is likely multifactorial and requires further in‐depth studies.

## 1 INTRODUCTION

Ewing sarcoma (ES) is the second most common malignant bone tumor affecting children, adolescents, and young adults (AYA).^1^ In the United States, ES is most frequently observed among non‐Hispanic White (NHW) males aged between 10 and 20 years, though there is an increasing recognition of cases among Hispanic patients.^2^ Although typically an osseous disease involving the pelvis or proximal long bones, ES can also manifest in soft tissues outside the bone structure. At a molecular level, ES is usually characterized by a distinctive chromosomal translocation, often involving the fusion of the EWSR1 gene on chromosome 22, with a known partner oncogene.^1^ While most patients present with localized disease, subclinical metastatic disease often lurks, contributing to a less favorable prognosis.

Remarkably, over the past four decades, the 5‐year overall survival (OS) rate for patients with ES has increased from <20% to >70%, due to advances in treatment approaches and supportive care. However, despite these significant strides, recurrence rates of nearly 25% continue to plague those with localized disease.^3^

The impact of racial and ethnic disparities on ES outcomes has been investigated on a national scale using the Surveillance, Epidemiology, and End Results (SEER) cancer surveillance data.^2^, ^4^, ^5^ These studies have highlighted increased mortality among Hispanic patients, non‐Hispanic Black (NHB) patients, and those residing in economically disadvantaged areas.^2^, ^5^, ^6^ Other population‐based ES studies performed outside of the SEER database showed that Hispanic patients with ES often experience a lower socioeconomic status (SES), reduced access to healthcare insurance, and diminished OS compared to their non‐Hispanic counterparts.^7^, ^8^ Lower SES has also been linked with poor OS, irrespective of racial or ethnic background.^7^, ^9^

The state of Florida does not participate in SEER but is home to a highly heterogeneous patient population. This diversity is partly due to the substantial presence of Hispanic and immigrant residents within the state, encompassing prominent subgroups such as Cubans, Puerto Ricans, Mexicans, Central Americans, South Americans, and Dominicans, each with populations exceeding 300,000. This contrasts with the remainder of the US, where more than 60% of Hispanics are of Mexican origin.^10^

With this unique demographic composition in Florida, our study has two specific objectives: first, to compare the incidence of ES in Florida with the nationally representative SEER population with a particular emphasis on the distinct Florida Hispanic population, and second, to examine racial and ethnic disparities in ES outcomes among patients in Florida. To achieve this, we leverage data from the comprehensive Florida Cancer Data System (FCDS) database.

## 2 METHODS

All patients newly diagnosed with histologically confirmed osseous Ewing Sarcoma between 2005 and 2018 were identified from both FCDS (*n* = 411) and SEER‐22 (*n* = 2732). To ensure accuracy, patients were selected based on primary site codes (C40.X and C41.X) and morphology codes (9260.3, and 9364.3‐9365.3) as per the International Classification of Diseases for Oncology, third edition (ICD‐O‐3). The FCDS, Florida's state cancer registry is highly complete and has been awarded with long‐standing Gold Certification status from the North American Association of Central Cancer Registries, a testament to its consistent data quality and completeness.^11^ While SEER data is publicly available (https://seer.cancer.gov/seerstat/started.html), datasets from FCDS can be requested with required approval from the Florida Department of Health Cancer Registry Program and Florida Department of Health Institutional Review Board (http://fcds.med.miami.edu/inc/datarequest.shtml). Data collected from both FCDS and SEER adhere to the same rigorous standards and encompass crucial variables such as age, race‐ethnicity, sex, insurance status, year of diagnosis, primary tumor site, tumor morphology, stage at diagnosis (localized, regional, and distant/metastatic), initial treatment regimen, and follow‐up for vital status.

We considered four mutually exclusive racial‐ethnic groups: Non‐Hispanic White (NHW), Non‐Hispanic Black (NHB), Hispanics (of any race), and a broader “Others” category. The latter included American Indian/Alaska Native individuals and those with multiracial backgrounds as well as NH‐Asian/Pacific Islanders due to the rarity of cases in these groups in Florida.

To compare the risk of ES between Florida and SEER, we computed incidence rates by racial‐ethnic group (NHW, NHB, and Hispanics) and sex, using age‐adjusted calculations according to the 2000 US standard population for both Florida and SEER‐22. Incidence rate ratios (IRR) with Tiwari 95% confidence intervals (CI) were calculated, with NHW as the reference group.

Temporal trends for osseus ES were studied and annual percent changes (APCs) for Florida and SEER‐22 were estimated for 2005–2018. This analysis was conducted using the Joinpoint regression program, version 4.9.1.^12^ The trends were fitted with 0 joinpoints, which were automatically selected for the best fit. T‐tests were employed to determine whether the APCs were statistically different from zero.

For survival analysis within Florida, variables included age (categorized as 0–14, 15–19, 20–24, 25–39, and 40+ years), sex (male and female), tumor site (limb, skull and mandible, thorax, spine, and pelvis), stage (SEER stage‐ localized, regional, or distant/metastatic), year of diagnosis (2005–2008, 2009–2012, 2013–2018), treatment received (surgery, radiation, and chemotherapy, each dichotomized as yes or no/unknown), poverty status (classified as very low, 0%–4.99% in poverty, 5%–9.99%, 10%–19.99%, and more than 20% based on percent poverty in the census tract of residence), and insurance status (private, non‐private [Medicare/Medicaid], and none).

To investigate survival disparities within Florida, we focused on the two largest racial‐ethnic groups (NHW and Hispanics), assessing bone cancer‐specific death as the outcome. Bone cancer‐specific survival was defined as the time elapsed in days from the date of ES diagnosis to whichever of the following two dates occurred first: the date of death for ES‐related fatalities or December 31, 2018 (for patients still alive), adhering to standard cancer registry protocols employing passive follow‐up, such as FCDS. Deaths unrelated to ES were censored at the time of death. Cause‐specific standards for bone cancer, as defined by SEER and dependent on the sequence number of ES, determined whether a death was attributed to the cancer.^13^

To facilitate interpretation, we converted survival time from days into years and months. We conducted both univariable and multivariable analyses employing Cox regression models to assess disparities by race/ethnicity while adjusting for age, sex, year of diagnosis, disease site, SEER staging as applicable, SES, and insurance type. Adjusted hazard ratios (aHR) with 95% CIs were calculated. Subsequent models were stratified by metastatic status. The proportional hazards assumption was assessed through analysis of the Schoenfeld residuals.

Our statistical analysis relied on SAS v9.4 software (SAS Institute Inc., Cary, NC). SEER data is publicly accessible, while we obtained exempt status from the Florida Department of Health's Institutional Review Board for the Florida cancer dataset.

## 3 RESULTS

### 3.1 Patient Characteristics

During the specified study period (2005–2018), a total of 2732 patients with ES were reported to the SEER‐22 database, while 411 cases were documented in the FCDS. Among the cases from SEER‐22, 60.6% (*n* = 1656) were male and 39.4% (*n* = 1076) were female. In Florida, 63.5% (*n* = 261) were male and 36.5% (*n* = 150) were female. Table 1 provides an overview of baseline characteristics for the Florida patients, with 60.8% identified as NHW, 28.7% as Hispanic, 6.3% as non‐Hispanic Black (NHB), and 4.1% as Asian, American Indian, or of mixed races.

**TABLE 1:** Demographics and clinical characteristics of patients with Ewing sarcoma (ES) by Race/ethnicity, Florida, 2005–2018.

Characteristics	Race, *N* (%)					*p*‐value
	Total	White	Black	Hispanic	All Others	
All patients	411 (100)	250 (60.8)	26 (6.3)	118 (28.7)	17 (4.1)	
Year of diagnosis						
2005–2008	102 (24.8)	56 (22.4)	<10	35 (29.7)	<10	0.68
2009–2012	142 (34.5)	88 (35.2)	<10	41 (34.7)	<10	
2013–2018	157 (40.6)	106 (42.4)	13 (50.0)	42 (35.6)	<10	
Sequence						
Single cancer	249 (60.6)	150 (60.0)	11 (42.3)	76 (64.4)	12 (70.6)	0.24
First of many cancers	81 (19.7)	54 (21.6)	<10	18 (15.3)	<10	
Not first of many cancers	81 (19.7)	46 (18.4)	<10	24 (20.3)	<10	
Age groups (years)						
0–14	137 (33.3)	77 (30.8)	10 (38.5)	44 (37.3)	<10	0.32
15–19	94 (22.9)	55 (22.0)	<10	30 (25.4)	<10	
20–24	57 (13.9)	37 (14.8)	<10	16 (13.6)	<10	
25–39	60 (14.6)	36 (14.4)	<10	18 (15.3)	<10	
40+	63 (15.3)	45 (18.0)	<10	10 (8.5)	<10	
Sex						
Male	261 (63.5)	156 (62.4)	13 (50.0)	84 (71.2)	<10	0.07
Female	150 (36.5)	94 (37.6)	13 (50.0)	34 (28.8)	<10	
SES by census						
Very low poverty (0%–4.9%)	64 (15.6)	49 (19.6)	<10	11 (9.3)	<10	<0.001
Low poverty (5%–9.9%)	103 (25.1)	66 (26.4)	<10	27 (22.9)	<10	
Medium poverty (0%–19.9%)	149 (36.3)	95 (38.0)	<10	41 (34.7)	<10	
High poverty (≥20%)	93 (22.6)	39 (15.6)	14 (53.8)	38 (32.2)	<10	
Unknown	<10	<10	<10	<10	<10	
Insurance						
No insurance	28 (6.8)	13 (5.2)	<10	11 (9.3)	<10	0.01
Non‐private insurance	171 (41.6)	88 (35.2)	14 (53.8)	60 (50.8)	<10	
Private insurance	212 (51.6)	149 (59.6)	<10	47 (39.8)	<10	
Tumor stage						
Localized	104 (25.3)	65 (26.0)	<10	31 (26.3)	<10	0.01
Regional	105 (25.5)	79 (31.6)	<10	19 (16.1)	<10	
Distant/metastatic	119 (29.0)	59 (23.6)	13 (50.0)	43 (36.4)	<10	
Unknown	83 (20.2)	47 (18.8)	<10	25 (21.2)	<10	
Location						
Limb	167 (40.6)	97 (38.8)	15 (57.7)	49 (41.5)	<10	0.62
Skull and mandible	21 (5.1)	13 (5.2)	<10	<10	<10	
Thorax	56 (13.6)	37 (14.8)	<10	14 (11.9)	<10	
Spine and pelvis	133 (32.4)	83 (33.2)	<10	40 (33.9)	<10	
Unknown	34 (8.3)	20 (8.0)	<10	11 (9.3)	<10	
Surgery						
Yes	208 (50.6)	134 (53.6)	<10	59 (50.0)	<10	0.13
No/unknown	203 (49.4)	116 (46.4)	18 (69.2)	59 (50.0)	10 (58.8)	
Radiation						
Yes	156 (38.0)	95 (38.0)	<10	49 (41.5)	<10	0.47
No/unknown	255 (62.0)	155 (62.0)	19 (73.1)	69 (58.5)	12 (70.6)	
Chemotherapy						
Yes	364 (88.6)	220 (88.0)	22 (84.6)	106 (89.8)	16 (94.1)	0.76
No/unknown	47 (11.4)	30 (12.0)	<10	12 (10.2)	<10	
Vital Status						
Alive/death other causes	281 (68.4)	181 (72.4)	17 (65.4)	72 (61.0)	11 (64.7)	0.17
Died of ES	130 (31.6)	69 (27.6)	<10	46 (39.0)	<10	

### 3.2 Incidence Rates and Trends

The age‐adjusted incidence rate (AAIR) of ES in NHW in Florida (males AAIR 2.5/1,000,000 95% CI [2.1–2.9 per 1,000,000], *n* = 156; females 1.5 [1.2–1.9], *n* = 93) was comparable to the rates among NHWs in SEER (males AAIR 2.1 95% CI [2.0–2.2], *n* = 1083; females 1.4 [1.2–1.5], *n* = 711) and constituted the majority of the cases of ES in both jurisdictions (Table 2).

**TABLE 2:** Age‐adjusted incidence (AAI) rates per 100,000 of Ewing sarcoma of the bone, Florida and SEER‐22, 2005–2018.

	FL		SEER‐22	
	N	AAI (95% CI)	N	AAI (95% CI)
Non‐Hispanic Whites				
Males	156	0.25 (0.21–0.29)	1083	0.21 (0.20–0.22)
Females	93	0.15 (0.12–0.19)	711	0.14 (0.13–0.16)
Non‐Hispanic Blacks				
Males	13	0.06 (0.03–0.10)	63	0.05 (0.04–0.06)
Females	13	0.06 (0.03–0.10)	37	0.02 (0.02–0.04)
Hispanics				
Males	84	0.26 (0.20–0.32)	382	0.13 (0.11–0.14)
Females	34	0.11 (0.08–0.15)	246	0.09 (0.08–0.10)
All races combined*				
Males	261	0.21 (0.19–0.24)	1656	0.16 (0.15–0.17)
Females	150	0.12 (0.10–0.15)	1076	0.11 (0.10–0.11)

* Includes Asians, American Indians and others.

**FIGURE 1:**
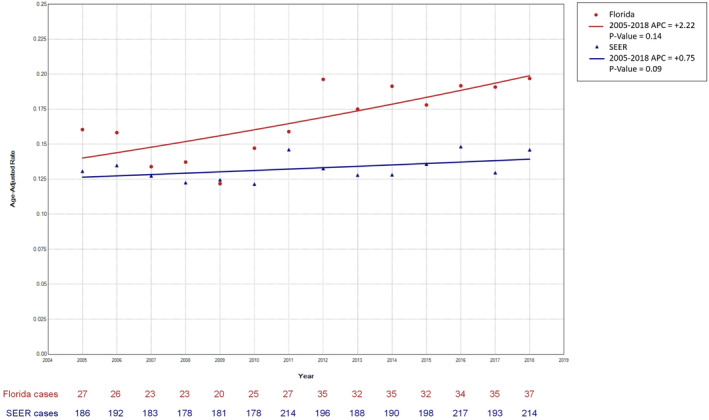
Florida and SEER‐22 trends of osseous Ewing sarcoma (all races combined), 2005–2018.

However, for Hispanic males, there was a significantly higher incidence of ES, approximately double, in Florida (AAIR 2.6/1,000,000; 95% CI [2.0–3.2 per 1,000,000; *n* = 84]) as compared to the SEER database (AAIR 1.3/1,000,000; 95% CI [1.1–1.4]; *n* = 382), *p* < 0.05. The Tiwari Incidence Rate Ratio (IRR) for Florida Hispanics versus SEER Hispanics significantly differed from 1: IRR = 2.05, 95%CI: 1.58–2.64. In contrast, for Hispanic females, the AAIRs were not significantly different between the two populations (Florida AAIR 1.1/1,000,000; (95% CI 0.8–1.5 per 1,000,000), *n* = 34; SEER AAIR 0.9/1,000,000 (0.8–1.0 per 1,000,000), *n* = 246) as shown in Table 2. From 2005 to 2018, the incidence of osseous ES remained stable in both Florida (APC: 2.22; 95% CI: −1.85 to 7.04; *p* = 0.14) and SEER‐22 (APC: 0.75; 95% CI: −0.50 to 2.02; *p* = 0.09) (Figure 1).

### 3.3 Impact of Race and Ethnicity on Cause‐specific Mortality

**FIGURE 2:**
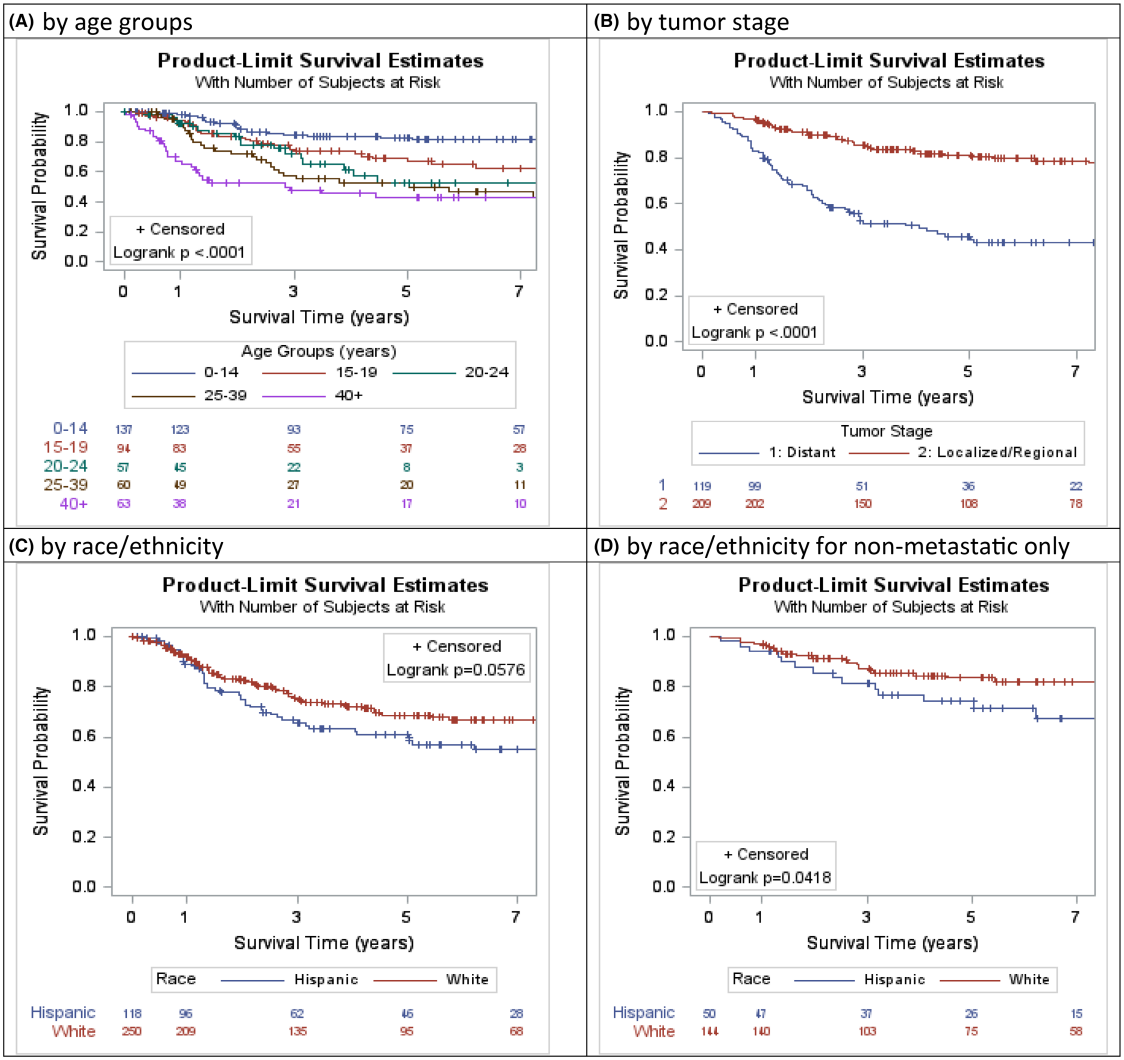
(A) By age groups, (B) by tumor stage, (C) by race/ethnicity, (D) by race/ethnicity for non‐metastatic patients only. Kaplan–Meier cause‐specific survival curves for patients with Ewing sarcoma in Florida (2005–2018).

The overall 5‐year cause‐specific survival for the Florida cohort was 65.4% (95%CI 60.3%–70.6%). Cox proportional hazards models included factors such as age, sex, year of diagnosis, site of disease, tumor stage, treatment received (including surgery, chemotherapy and radiation), SES‐poverty status, and insurance type (Table 3). On univariable analysis, those with older age, distant stage at diagnosis, no chemotherapy, and no surgical resection were associated with a lower survival compared to younger age (Figure 2A), localized/regional stage at diagnosis (Figure 2B), and those receiving chemotherapy or surgical resection (Table 3) respectively. For all stages combined, the cancer‐specific mortality for Hispanics (aHR 1.43; 95%CI: 0.99–2.08) was not significantly different from NHWs, *p* = 0.06 (Figure 2C).

**TABLE 3:** Demographics, clinical characteristics and cause‐specific survival analysis for patients with Ewing sarcoma in Florida, 2005–2018.

	*N*	Univariable		Multivariable	
		HR (95%CI)	*p*‐value	HR (95%CI)	*p*‐value
Age (years)					
0–14	137	1 [Reference]		1 [Reference]	
15–19	94	1.96 (1.14, 3.37)	0.15	1.85 (1.07, 3.21)	0.03
20–24	57	2.62 (1.42, 4.86)	0.002	2.45 (1.31, 4.57)	0.01
25–39	60	3.32 (1.91, 5.65)	<0.001	2.90 (1.63, 5.17)	<0.001
>40	63	4.88 (2.87, 8.30)	<0.001	4.69 (2.65, 8.30)	<0.001
Sex					
Male	261	1 [Reference]			
Female	150	1.00 (0.69, 1.43)	0.98		
Race/Ethnicity					
Non‐Hispanics White	250	1 [Reference]		1 [Reference]	
Non‐Hispanic Blacks	26	1.43 (0.72, 2.87)	0.31	1.05 (0.52, 2.14)	0.89
Hispanics	118	1.43 (0.99, 2.08)	0.06	1.32 (0.90, 1.94)	0.16
All others	17	1.27 (0.55, 2.92)	0.58	1.09 (0.47, 2.54)	0.84
Location					
Limb	167	1 [Reference]			
Skull and mandible	21	0.42 (0.13, 1.34)	0.14		
Spine and pelvis	133	1.16 (0.78, 1.73)	0.48		
Thorax	56	0.70 (0.36, 1.34)	0.28		
Unknown	34	2.44 (1.46, 4.10)	<0.001		
Year of diagnosis					
2005–2008	102	1 [Reference]			
2009–2012	142	1.14 (0.76, 1.71)	0.54		
2013–2018	167	0.98 (0.60, 1.59)	0.93		
Tumor stage					
Localized	104	1 [Reference]		1 [Reference]	
Regional	105	1.24 (0.68, 2.24)	0.48	1.62 (0.88, 2.96)	0.12
Distant/metastatic	119	3.64 (2.20, 6.04)	<0.001	3.70 (2.20, 6.21)	<0.001
Unknown	83	3.54 (1.95, 6.43)	<0.001	2.60 (1.41, 4.80)	0.002
Chemotherapy					
Yes	364	1 [Reference]		1 [Reference]	
No/unknown	47	2.02 (1.29, 3.17)	0.01	1.29 (0.79,2.11)	0.30
Surgery					
Yes	208	1 [Reference]		1 [Reference]	
No/unknown	203	2.30 (1.60, 3.31)	<0.001	1.80 (1.24, 2.63)	0.002
Radiation					
Yes	156	1 [Reference]			
No	255	1.01 (0.71, 1.44)	0.95		
SES by census					
Very low poverty (0%–4.9%)	64	1 [Reference]			
Low poverty (5%–9.9%)	103	1.09 (0.63, 1.88)	0.77		
Medium poverty (0%–19.9%)	149	0.86 (0.51, 1.45)	0.56		
High poverty (≥20%)	93	0.97 (0.55, 1.72)	0.93		
Unknown	2	1.07 (0.14, 7.94)	0.95		
Insurance					
Private	212	1 [Reference]			
Non‐private Insurance	171	1.02 (0.71, 1.45)	0.90		
None/Unknown	28	0.96 (0.48, 1.93)	0.92		

Only variables that were significant in the univariable analysis (*p* ≤ 0.05) were included in the final multivariable analyses. For all patients, those aged 40 and older had a 4.69‐fold higher risk of death (aHR 4.69, 95%CI: 2.65–8.30, *p* < 0.001) compared to ES patients younger than 15. Patients diagnosed with distant metastatic disease had a 3.70‐fold higher risk of death (95%CI: 2.20–6.21, *p* < 0.001) compared to those diagnosed in localized stage. Additionally, those who did not receive surgery had a 1.80‐fold higher risk of death (aHR 1.80, 95%CI: 1.24–2.63, *p* = 0.002) compared to those who did. Survival did not significantly differ based on race‐ethnicity, poverty level or insurance status for patients with ES in Florida, as detailed in Table 3.

However, among the restricted subset of nonmetastatic ES (*n* = 209), Hispanics exhibited a lower survival (Figure 2D) and an increased risk of death due to ES (aHR 2.32; 95%CI: 1.20–4.46; *p* = 0.012) compared to NHWs, after adjusting for all other variables, as shown in Table 4.

**TABLE 4:** Demographics, clinical characteristics and cause‐specific survival analysis for patients with nonmetastatic Ewing sarcoma in Florida (localized or regional), 2005–2018 multivariable analysis.

	*N*	HR (95%CI)	*p*‐value
Age (years)			
0–14	81	1 [Reference]	
15–19	48	1.20 (0.48, 3.02)	0.70
20–24	26	4.07 (1.56, 10.57)	0.004
25–39	30	3.29 (1.37, 7.90)	0.01
>40	24	4.14 (1.53, 11.24)	0.01
Race/ethnicity			
Non‐Hispanic Whites	144	1 [Reference]	
Non‐Hispanic Blacks	8	0.73 (0.10, 5.46)	0.76
Hispanics	50	2.32 (1.20,4.46)	0.01
All others	7	1.26 (0.29, 5.51)	0.76
Stage			
Localized	104	1 [Reference]	0.03
Regional	105	2.06 (1.09, 3.88)	
Surgery			
Yes	126	1 [Reference]	0.04
No/unknown	83	1.91 (1.04, 3.51)	

*Note*: sex, receipt of radiation therapy, poverty level, insurance type, tumor location, year of diagnosis were not significant at alpha = 0.05.

## 4 DISCUSSION

Ewing sarcoma is typically more prevalent in males, aged 10 to 20 years, and has been primarily associated with NHW^1^ populations, with significant disparities in survival based on race‐ethnicity. However, our study, conducted in the ethnically diverse state of Florida, uncovers significant findings. Notably, Hispanic males in Florida displayed significantly higher incidence rates of ES than their counterparts in the SEER population during the years 2005–2018, though incidence rates among female Hispanics did not differ between Florida and SEER. Moreover, our analysis of data from the FCDS reaffirms a generally worse prognosis for nonmetastatic ES among Hispanic patients, of both sexes, when compared to NHW individuals within Florida.

Previous studies analyzing incidence rates and survival among Hispanic patients with ES have predominantly relied on SEER registries.^14^ The SEER program is a crucial resource for cancer‐related epidemiological and healthcare service data, currently encompassing 50% of the US population through 22 geographically diverse population‐based cancer registries.^15^ Despite the recent expansion of the SEER program to include additional registries in states with substantial Hispanic populations, such as New York and Texas, it notably lacks representation from Florida, despite being the third most populous state.^16^ Moreover, Florida's Hispanic population is distinctively different from the rest of the US as it predominantly consists of Cubans (32%) and Puerto Ricans (18%) which differs from an overall majority Mexican population in other states with large Hispanic Populations included in SEER, such as Texas and California.^17^ It is important to recognize the diversity within this growing ethnic group, given that cancer is the second leading cause of mortality among Hispanics. Higher mortality rates have also been demonstrated among Cubans and Puerto Ricans with various cancers, including endometrial, prostate, and colorectal cancer, compared to NHW population, which also underscores the importance of including these populations in comprehensive cancer databases.^17^, ^18^ Similar in‐depth studies for sarcomas are currently lacking and it is unclear if the survival disparity noted in our study is the result of this heterogeneity among the Hispanics.

### 4.1 Incidence

Our study highlights the increased incidence of ES among Hispanic males in Florida, which may be a result of the underlying diversity within this ethnic group and warrants further investigation. From an epidemiologic context, immigrants tend to gradually mimic the disease profile of the host population, thus studies investigating disparities need to account for geographic origin as well as length of stay as potential contributory factors.^19^ However, this phenomenon has not been explored in cancers that affect predominantly older children and AYA such as ES.

Some studies have suggested that ancestry‐associated genetic variation may play a larger role in sarcoma etiology as compared to the SES‐related factors.^20^ For ES specifically, distinct polymorphisms were noted in the *NR0B1* gene for patients of African and European descent, potentially influencing EWS/FLI‐mediated gene expression and thereby oncogenesis.^21^ The African patients were noted to have >30 GGAA‐microsatellite repeats which were thought to possibly explain the substantially lower incidence of ES in black individuals as compared to white. Furthermore, a genome wide association study (GWAS) in European patients, identified more haplotype frequencies in the candidate risk loci, which were less prevalent in the African patients.^22^ Similar genetic studies for Hispanic patients are deficient. Exploring these possible genetic variations could shed some insight to the notable difference in incidence of ES among Hispanic individuals.

### 4.2 Cause‐specific survival

Age, tumor size, site of disease, and metastatic status have independently been identified as risk factors influencing OS in patients with ES, leading to the creation of survival prediction models based on SEER data.^15^ Like in other studies, older age, lack of surgical resection and presence of distant metastatic disease were also shown to have a significant impact on cause‐specific survival in our study.

In the multivariable analysis of disparities within Florida, cause‐specific survival was worse for Hispanics compared to NHW for nonmetastatic disease. This is consistent with the existing literature demonstrating worse outcomes for white Hispanic, Black and Asian patients compared to Non‐Hispanic patients with ES, when stratified by metastatic status.^2^ In their analysis, Worch et al, controlled for age, year of diagnosis, pelvic site, and tumor size in addition to metastatic status, with continued demonstration of significantly higher hazard ratio for mortality among white Hispanics as compared to NHW (HR = 1.46, *p* = 0.023).

According to the same study, ES patients other than NHW are more likely to present with larger tumors (*p* < 0.042) and soft tissue involvement (*p* < 0.0001) rather than bone tumors.^2^ In our study, we included only patients with osseus ES and still noted a higher cause‐specific mortality in Hispanics as compared to NHW in Florida. Another study reported an association between Hispanic ethnicity and metastatic disease at presentation in Osteosarcoma, however a similar association for ES was not seen when early diagnosis years were excluded.^6^

The poorer outcomes experienced by Hispanics may result from a combination of non‐biological and biological factors.^2^ Hispanics typically have lower SES, reduced access to healthcare, and are less likely to have insurance when compared to NHW populations.^8^ Hispanic patients are also more likely to present with initial symptoms to the emergency department compared to non‐Hispanic patients who are more likely to seek care from primary care providers.^8^

The National Healthcare Statistics American Trends Panel (ATP) 2021 showed that Hispanics aged 0–65 years were often uninsured (one quarter) or had Medicaid/CHIP (one third).^16^ Approximately half of Hispanics reported reduced access to quality medical care, and 44% identified multiple communication barriers to care, whether it may be language or culturally based.^23^ Reduced access to care and lower SES typically can lead to delayed presentation of disease, delayed treatment of disease, overall decreased participation in clinical trials and therefore worse outcomes in ES. A study utilizing the SEER database to evaluate all bone sarcomas noted higher risk of metastatic disease at presentation for those Medicaid or no insurance when compared with those with private health insurance.^4^ Insurance status and SES were not found to be statistically significant in our study. Even after adjusting for age and stage, Hispanic patients with nonmetastatic ES fared worse than NHW patients, emphasizing the need for further investigation. Additionally, Hispanic ethnicity has been shown to be an independent predictor of poor outcomes in pediatric cancers, though further studies are needed to understand this disparity.^8^

While biologic factors have been implicated for outcome differences for some of the other cancers such acute lymphoblastic leukemia, lymphomas,^24^ their role in sarcomas remains unclear and warrants exploration. Our institutional study evaluating genetic alterations in 174 patients with sarcoma noted a higher incidence of actionable mutations in non‐Hispanic patients with sarcoma when compared with Hispanic patients (62% vs. 38%).^25^ ES is a fusion driven sarcoma with less genetic heterogeneity among patients to explain the survival differences. In a two‐institution limited study, biologic factors such as basic tumor histopathology as well as posttreatment necrosis and radiographic objective responses were not noted to be significantly different for the Latino/Hispanic versus non‐Latino/Hispanic patients with ES.^8^ However, a GWAS may be more useful in identifying factors that explain these outcome differences despite adjustment for SES. In addition, pharmacogenomic studies also deserve further investigation, and could potentially be driving or contributing to the observed disparities.

### 4.3 Limitations

Our study is not without limitations. While SEER and FCDS data are fully population‐based, thereby reducing the potential for bias from selection factors typical of hospital‐based studies, they may lack certain clinically relevant details, such as next generation sequencing data, comprehensive treatment information, co‐morbidities, extent of surgical resection, surgical margins, and toxicities. This limitation underscores the importance of integrating multiple data sources for comprehensive clinical characterization of ES. Additionally, possible misclassification of ethnicity and cancer diagnosis in registry records may also have occurred. Data on birthplace in registries is incomplete, so the distinction between foreign‐born and US‐born Hispanics was not possible to analyze in our study. Moreover, the predominantly White Cuban American population in Florida may differ significantly from other Hispanic subgroups in terms of genetic ancestry, potentially distorting comparisons.^19^

## 5 CONCLUSION

This study represents the most comprehensive and up‐to‐date analysis of ethnic disparities in ES incidence and outcomes in the large and diverse state of Florida. It highlights the unexpectedly higher ES incidence among Hispanic males in Florida compared to the broader US population, along with remarkably worse survival for nonmetastatic disease among Hispanics of both sexes compared to NHW individuals. These disparities likely result from a complex interplay of factors and merit further in‐depth investigation.

## AUTHOR CONTRIBUTIONS

**Aditi Dhir:** Conceptualization (equal); data curation (equal); formal analysis (equal); investigation (equal); methodology (equal); project administration (equal); resources (equal); software (equal); supervision (equal); validation (equal); visualization (equal); writing – original draft (equal); writing – review and editing (equal). **Rachna Rahul:** Data curation (equal); formal analysis (equal); investigation (equal); methodology (equal); project administration (equal); resources (equal); software (equal); validation (equal); visualization (equal); writing – original draft (equal); writing – review and editing (equal). **Dan Pham:** Data curation (equal); formal analysis (equal); investigation (equal); methodology (equal); project administration (equal); resources (equal); software (equal); validation (equal); visualization (equal); writing – original draft (equal); writing – review and editing (equal). **Rachel Kronenfeld:** Data curation (equal); formal analysis (equal); funding acquisition (equal); investigation (equal); methodology (equal); project administration (equal); resources (equal); software (equal); supervision (equal); validation (equal); visualization (equal); writing – original draft (equal); writing – review and editing (equal). **Tulay Koru‐Sengul:** Data curation (equal); formal analysis (equal); funding acquisition (equal); investigation (equal); methodology (equal); project administration (equal); resources (equal); software (equal); supervision (equal); validation (equal); visualization (equal); writing – original draft (equal); writing – review and editing (equal). **Paulo S. Pinheiro:** Conceptualization (equal); data curation (equal); formal analysis (equal); funding acquisition (equal); investigation (equal); methodology (equal); project administration (equal); resources (equal); software (equal); supervision (equal); validation (equal); visualization (equal); writing – original draft (equal); writing – review and editing (equal). **Qinran Liu:** Data curation (equal); formal analysis (equal); investigation (equal); methodology (equal); project administration (equal); resources (equal); software (equal); validation (equal); visualization (equal); writing – original draft (equal); writing – review and editing (equal).

## CONFLICT OF INTEREST STATEMENT

The authors have no conflict of interest to declare.

## Data Availability

For FCDS, the datasets are available by request with required approvals from the Florida Department of Health Cancer Registry Program and Florida Department of Health Institutional Review Board. Applications for data request are available from the FCDS Webpage (http://fcds.med.miami.edu/inc/datarequest.shtml). For SEER: data is publicly available from: https://seer.cancer.gov/seerstat/started.html.
